# Supplementary material for: Botryllin, a Novel Antimicrobial Peptide from the Colonial Ascidian Botryllus schlosseri
Source: Mar Drugs. 2023 Jan 21;21(2):74. doi: 10.3390/md21020074 (PMC9966394; doi:10.3390/md21020074)
Supplement: Supplementary file 1 [file marinedrugs-21-00074-s001.zip › marinedrugs-2139077-supplementary.pdf]

Supplementary Information

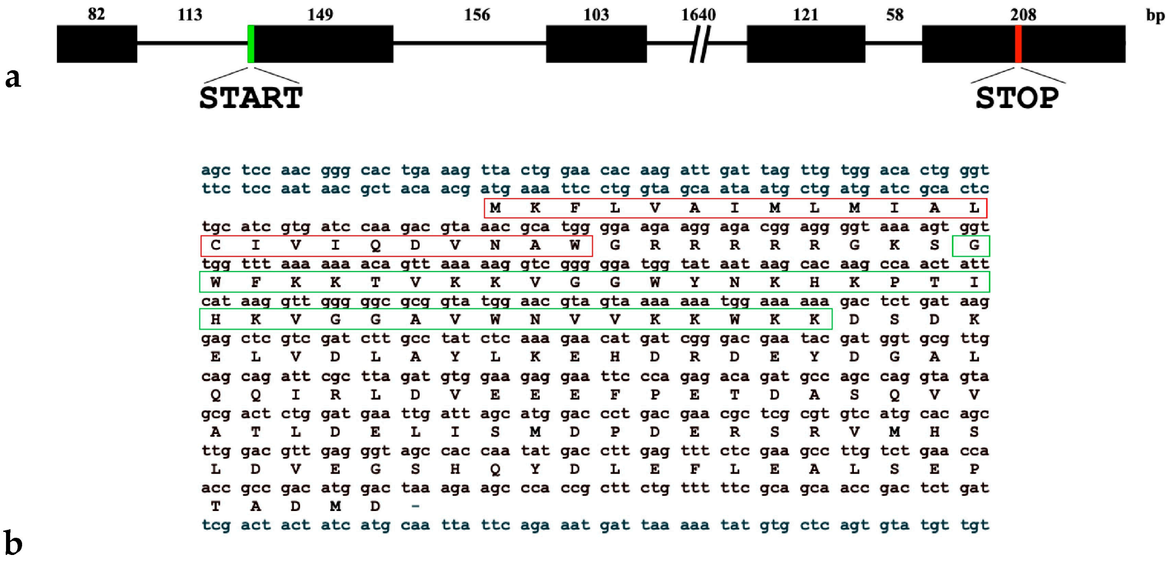

Figure S1. (**a**) Gene organisation of botryllin. (**b**) Nucleotide and amino acid sequences of the pre-propeptide transcript. The signal peptide and the mature peptide are boxed red and green, respectively.
